# Pivotal role of IL-8 derived from the interaction between osteosarcoma and tumor-associated macrophages in osteosarcoma growth and metastasis via the FAK pathway

**DOI:** 10.1038/s41419-024-06487-y

**Published:** 2024-02-01

**Authors:** Rikito Tatsuno, Jiro Ichikawa, Yoshihiro Komohara, Cheng Pan, Tomonori Kawasaki, Atsushi Enomoto, Kaoru Aoki, Keiko Hayakawa, Shintaro Iwata, Takahiro Jubashi, Hirotaka Haro

**Affiliations:** 1 Department of Orthopaedic Surgery, University of Yamanashi, Yamanashi, Japan; 2 Department of Cell Pathology, Graduate School of Medical Sciences, Kumamoto University, Kumamoto, Japan; 3 Department of Pathology, Saitama Medical University International Medical Center, Saitama, Japan; 4 Department of Pathology, Nagoya University Graduate School of Medicine, Aichi, Japan; 5 Physical Therapy Division, School of Health Sciences, Shinshu University, Nagano, Japan; 6 Department of Orthopaedic Oncology, Cancer Institute Hospital of Japanese Foundation for Cancer Research, Tokyo, Japan; 7 Department of Musculoskeletal Oncology and Rehabilitation, National Cancer Center Hospital, Tokyo, Japan

**Keywords:** Bone cancer, Cancer microenvironment

## Abstract

The prognosis of osteosarcoma (OS) has remained stagnant over the past two decades, requiring the exploration of new therapeutic targets. Cytokines, arising from tumor-associated macrophages (TAMs), a major component of the tumor microenvironment (TME), have garnered attention owing to their impact on tumor growth, invasion, metastasis, and resistance to chemotherapy. Nonetheless, the precise functional role of TAMs in OS progression requires further investigation. In this study, we investigated the interaction between OS and TAMs, as well as the contribution of TAM-produced cytokines to OS advancement. TAMs were observed to be more prevalent in lung metastases compared with that in primary tumors, suggesting their potential support for OS progression. To simulate the TME, OS and TAMs were co-cultured, and the cytokines resulting from this co-culture could stimulate OS proliferation, migration, and invasion. A detailed investigation of cytokines in the co-culture conditioned medium (CM) revealed a substantial increase in IL-8, establishing it as a pivotal cytokine in the process of enhancing OS proliferation, migration, and invasion through the focal adhesion kinase (FAK) pathway. In an in vivo model, co-culture CM promoted OS proliferation and lung metastasis, effects that were mitigated by anti-IL-8 antibodies. Collectively, IL-8, generated within the TME formed by OS and TAMs, accelerates OS proliferation and metastasis via the FAK pathway, thereby positioning IL-8 as a potential novel therapeutic target in OS.

## Introduction

Osteosarcoma (OS) is the most prevalent primary bone tumor afflicting children and adolescents [1]. Although advances in chemotherapy and surgical interventions have gradually enhanced OS treatment outcomes, and the five-year survival rate has increased to approximately 70%, the prognosis for patients with lung metastases, particularly those occurring during chemotherapy remains exceptionally poor [2]. Consequently, the development of novel chemotherapeutic agents, as well as elucidating the mechanism of lung metastasis, is essential.

In sarcomas, similar to carcinomas, the tumor microenvironment (TME), including components such as the extracellular matrix, platelets, fibroblasts, lymphocytes, bone marrow-derived inflammatory cells, and signaling molecules, has garnered attention [3]. Tumor-associated macrophages (TAMs), a major TME component [4], can be broadly classified into M1, which is primarily pro-inflammatory, and M2, which is tumor-promoting [5]. Additionally, various TAM phenotypes, including in sarcomas [6], has been documented, highlighting their multifaceted roles in various processes, including tumor growth, metastasis promotion, and angiogenesis. TAM-derived cytokines play a central role in these processes [7]. Although we reported the significance of IL-6 in undifferentiated pleomorphic sarcoma (UPS) [8], the critical cytokines in OS remain inadequately understood.

IL-8 induces chemotaxis of neutrophils in inflamed tissues and promote proliferation and angiogenesis in tumors [9]. IL-8 serves as a metastatic and prognostic factor in cancer [10], with a possible parallel in vitro and in vivo effect in OS [11]. However, the mechanisms governing IL-8 production in the TME and its influence on OS remain unclear. Therefore, we aimed to comprehensively elucidate the role of IL-8, derived from the interaction between OS and TAMs, and its impact on OS growth and metastasis.

## Results

### TAMs exhibit tumor-promoting activity in OS patient samples

**Fig. 1:**
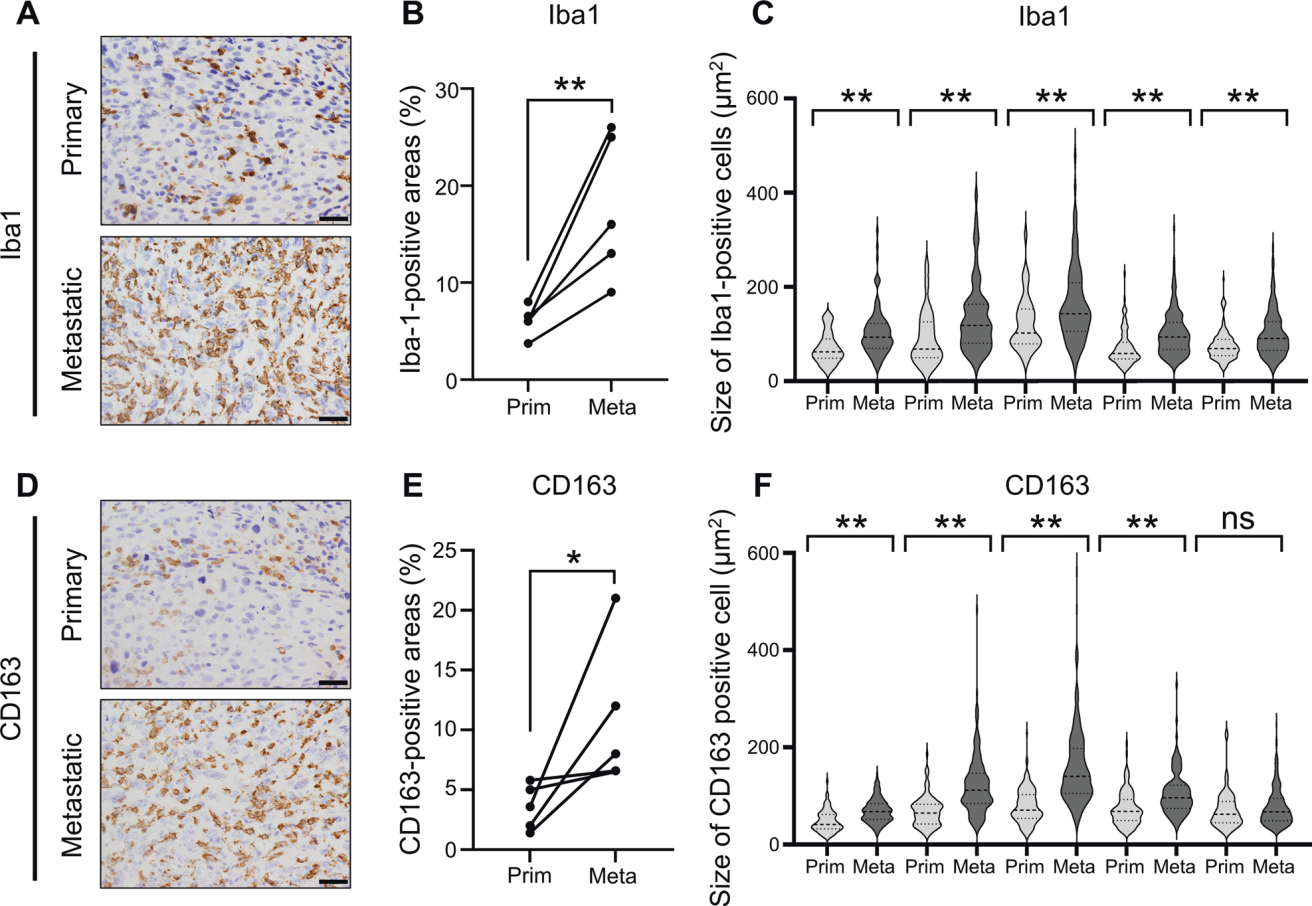
Expression and quantification of TAM in human osteosarcoma tissues from primary and lung metastasis sites. **A** Immunohistochemical analysis of Iba1-positive cells in primary tumors and lung metastases of patients with OS and lung metastases. Scale bars represent 50 μm. **B** Quantification of Iba1-positive areas per high-power fields (400× magnification). **C** Violin plot depicting Iba1-positive cells in primary and lung metastases from each sample (N = 5). **D** Immunohistochemical analysis of CD163-positive cells in primary tumors and lung metastases of OS patients with lung metastases. Scale bars represent 50 μm. **E** Quantification of CD163-positive areas per high-power fields (×400 magnification). **F** Violin plot illustrating CD163-positive cells in primary and lung metastases from each sample (N = 5). For the analysis of infiltrating macrophages, ten non-overlapping high-power fields (400× magnification) were randomly selected in tumor areas and the proportion (%) of positive areas was evaluated using the ImageJ software. For cell size analysis, 100–150 cells per sample that had their nucleus in the pictures were encircled, and the size of encircled area was measured based on the scale bar. ns: not significant, OS: osteosarcoma. Data are presented as mean ± standard deviation. *p < 0.05 and **p < 0.01.

The role of macrophages in OS was elucidated by comparing TAM quantities in primary and lung metastatic OS sites in patients with lung metastases using immunohistochemistry (IHC). Details of the patient background are presented in Table 1. The numbers and sizes of cells positive for Iba1, a macrophage marker, and CD163, an M2 macrophage/protumor marker, were notably higher in lung metastases than that in the primary tumor (Fig. 1A–F, Supplemental Fig. 1A). These cells frequently and highly co-expressed both markers (Supplemental Fig. 1B), suggesting that TAMs can potentially support OS progression.

**Table 1:** Clinical characteristics of patients with osteosarcoma.

Patient	Age (years)	Gender	Site	Diagnosis	Neoadjuvant chemotherapy	Adjuvant chemotherapy	Duration from diagnosis until metastasis (months)	Duration from operation until metastasis (months)	Grade^a^	Local recurrence	Status
OS 1	19	Female	Femur	OB	Yes	Yes	16	12	2	No	AWD
OS 2	16	Male	Femur	OB	Yes	Yes	24	18	2	No	NED
OS 3	12	Male	Femur	OB	Yes	Yes	9	4	1	No	NED
OS 4	13	Male	Tibia	OB	Yes	Yes	18	14	2	No	DOD
OS 5	15	Female	Femur	OB	Yes	Yes	3	9	1	No	DOD
Mean	15.0						14.0	11.4			

*AWD* alive with disease, *DOD* dead of disease, *NED* no evidence of disease, *OB* osteoblastic osteosarcoma.

^a^Histological necrosis after neoadjuvant chemotherapy: Grade 0, 0–50%; Grade 1, 51–90%; Grade 2, 91–99%; Grade 3, 100%.

### Cytokines derived from OS and macrophage co-culture exert tumor-promoting effects

**Fig. 2:**
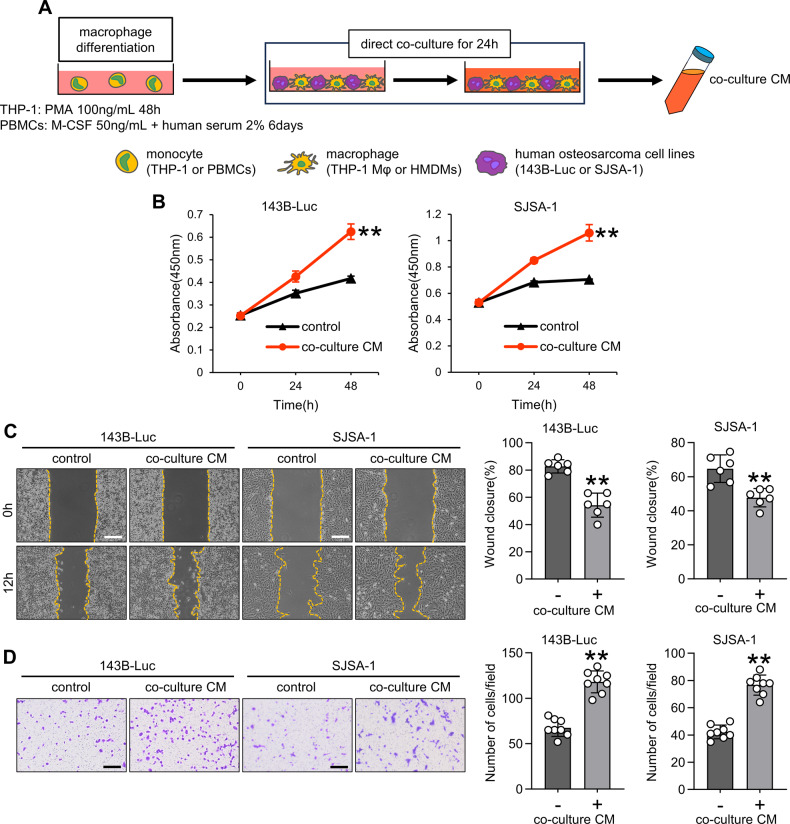
Effect of co-culture CM on proliferation, migration, and invasion in osteosarcoma cells. **A** Schematic diagram illustrating the method for differentiating monocytes into macrophages and co-culturing them with OS cells. **B** Cell viability assay for OS cells treated with or without co-cultured CM using the Cell Counting Kit-8 assay. **C** and **D** Scratch and invasion assays for OS cells treated with or without co-culture CM. The scratch assay was conducted for 12 h, and the invasion assay was performed for 24 h. Scale bars represent 500 μm. CM conditioned medium, HMDMs human monocyte-derived macrophages, OS osteosarcoma, PBMCs peripheral blood mononuclear cells, PMA phorbol 12-myristate 13-acetate, THP-1 Mφ THP-1-derived macrophage. Data are presented as mean ± standard deviation. **p < 0.01. All data were obtained from at least three independent experiments.

OS and macrophages were co-cultured to recapitulate the TME (Fig. 2A). WST, scratch, and invasion assay results revealed that co-culture CM derived from OS and THP-1-derived macrophages promoted OS proliferation, migration, and invasion (Fig. 2B–D), highlighting the OS-promoting effect of cytokines produced from the co-culture of OS and macrophages.

### IL-8 is the primary cytokine produced from the OS and macrophage co-culture

**Fig. 3:**
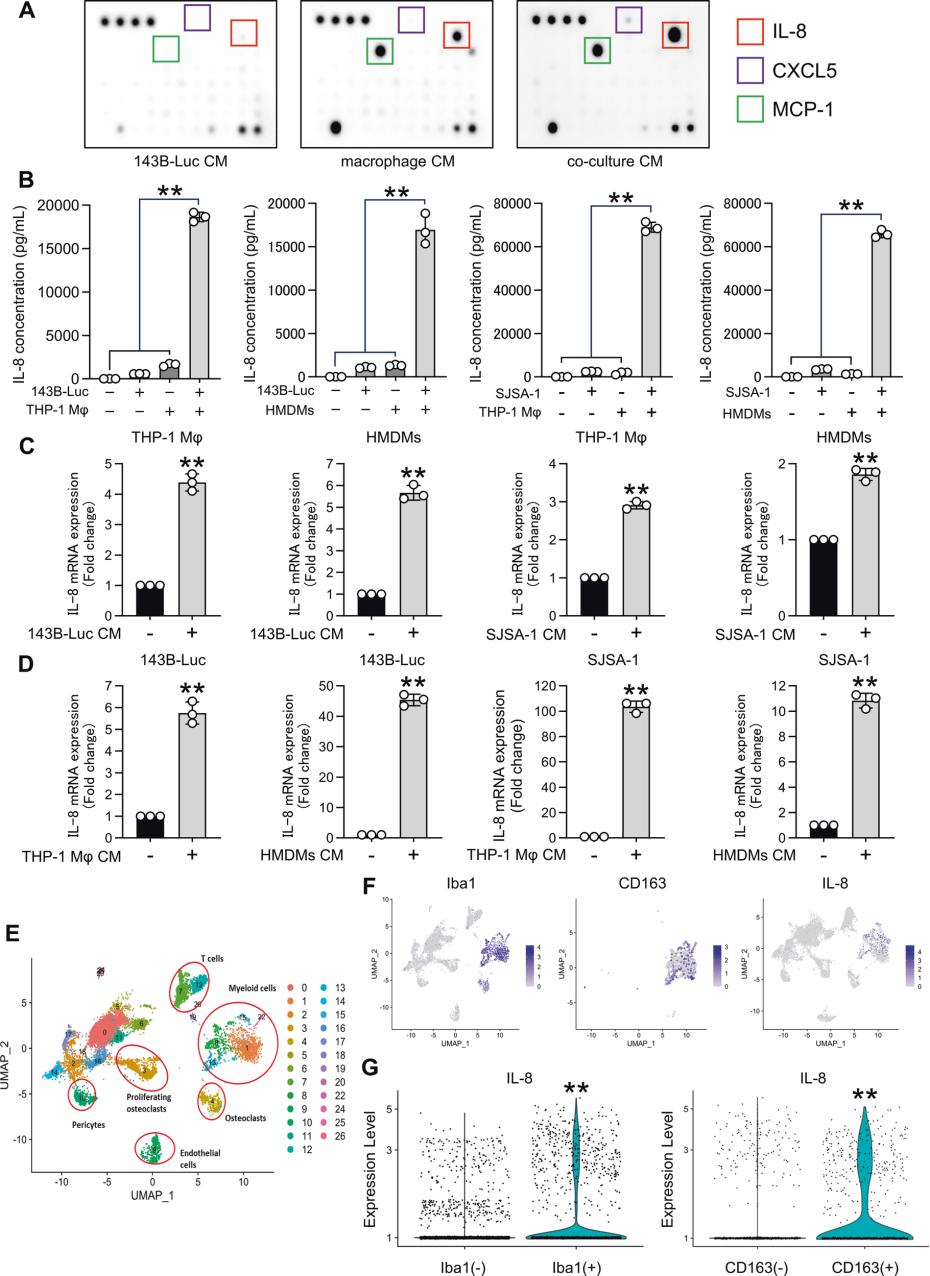
Interaction between osteosarcoma and macrophages increased IL-8 production. **A** Cytokine arrays of 143B-Luc cells, human macrophages, and co-culture CM. **B** IL-8 production during the co-culture of OS cells (143B-Luc, SJSA-1) and macrophages (THP-1 Mφ, HMDMs). **C** IL-8 expression in macrophages stimulated with OS-CM. **D** IL-8 expression in OS cells stimulated with macrophage CM. **E** UMAP plot of OS lung metastases. **F** UMAP plot of Iba1, CD163, and IL-8 expression in myeloid cell clusters. **G** Violin plot depicting IL-8 expression in Iba1^+/−^ or CD163^+/−^ myeloid cell clusters. CM conditioned medium, HMDMs human monocyte-derived macrophages, OS osteosarcoma, THP-1 Mφ THP-1-derived macrophage. Data are presented as mean ± standard deviation. **p < 0.01. All data were obtained from at least three independent experiments.

We investigated the precise mechanisms underlying the promoting effects of co-culture CM derived from OS and macrophages by monitoring the cytokine profiles using a cytokine array and observed alterations in several cytokines, including CXCL5 and MCP-1. Notably, IL-8 levels were substantially increased in co-culture CM than that in the supernatant of individual OS and macrophage cultures (Fig. 3A). Further investigation into IL-8 production in co-culture involving 143B-Luc or SJSA-1 cells with macrophages revealed increased IL-8 production when OS cells were co-cultured with either human or THP-1-derived macrophages (Fig. 3B). Furthermore, addition of OS-CM to THP-1-derived or human macrophages (Supplemental Fig. 2B) increased the IL-8 gene and protein expression (Fig. 3C, Supplemental Fig. 2C). Notably, OS cells themselves also produced IL-8 (Supplemental Fig. 2A), and when macrophage CM was introduced to OS cells (Supplemental Fig. 2B), it similarly increased IL-8 gene and protein expression, mirroring the effects of OS-CM addition to macrophages (Fig. 3D, Supplemental Fig. 2D). Furthermore, single-cell RNA-seq analysis of OS patients revealed higher IL-8 expression in Iba1^+^CD163^+^ TAMs than that in Iba-1^−^CD163^−^ TAMs (Fig. 3E–G). These results suggest that within the OS TME, TAMs interact with OS to enhance IL-8 production.

### IL-8 in co-culture CM promotes OS proliferation, migration, and invasion

**Fig. 4:**
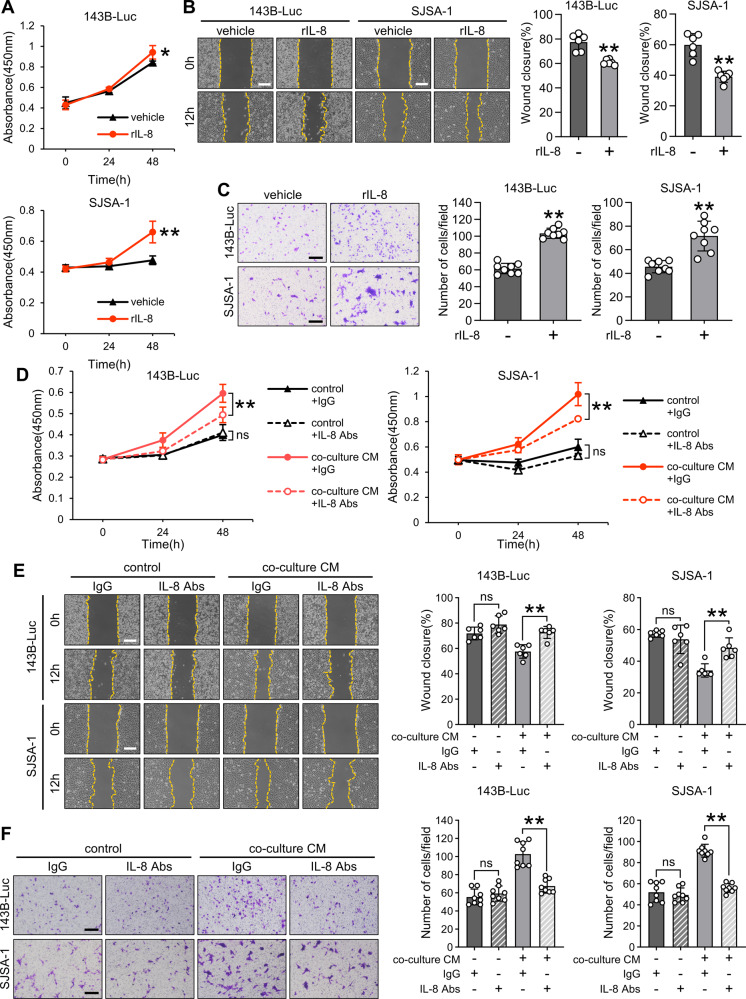
Effect of IL-8 in co-culture CM on proliferation, migration, and invasion of osteosarcoma cells. **A** Cell viability assay for OS cells treated with or without recombinant IL-8 (rIL-8, 10 ng/mL) using Cell Counting Kit-8. **B**, **C** Scratch and invasion assays for OS cells treated with or without co-culture CM. The scratch assay was performed for 12 h, and the invasion assay was conducted for 24 h. Scale bars represent 500 μm. **D** Cell viability assay for OS cells treated with or without co-culture CM and with or without anti-IL-8 antibodies (IL-8 Abs, 1 µg/mL) using Cell Counting Kit-8. **E**, **F** Scratch and invasion assays for OS cells treated with or without co-culture CM and with or without IL-8 Abs (100 ng/mL). The scratch assay was performed for 12 h, and the invasion assay was conducted for 24 h. Scale bars represent 500 μm. CM: conditioned medium, OS: osteosarcoma, ns: not significant. Data are presented as mean ± standard deviation. *p < 0.05 and **p < 0.01. All data were obtained from at least three independent experiments.

To elucidate the effects of IL-8 within co-culture CM on OS proliferation, migration, and invasion, we confirmed that OS cells express CXCR1 and CXCR2, which are IL-8 receptors (Supplemental Fig. 3A). Furthermore, recombinant IL-8 exhibited a similar trend in promoting proliferation, migration, and invasion in both OS cell types (Fig. 4A–C). Inhibiting IL-8 in co-culture CM using an anti-IL-8 antibody attenuated the proliferation, migration, and invasion, which were promoted by co-culture CM (Fig. 4D–F). Thus, IL-8 within co-culture CM plays a role in augmenting OS proliferation, migration, and invasion.

### IL-8 promotes OS proliferation, migration, and invasion via the focal adhesion kinase (FAK) pathway

**Fig. 5:**
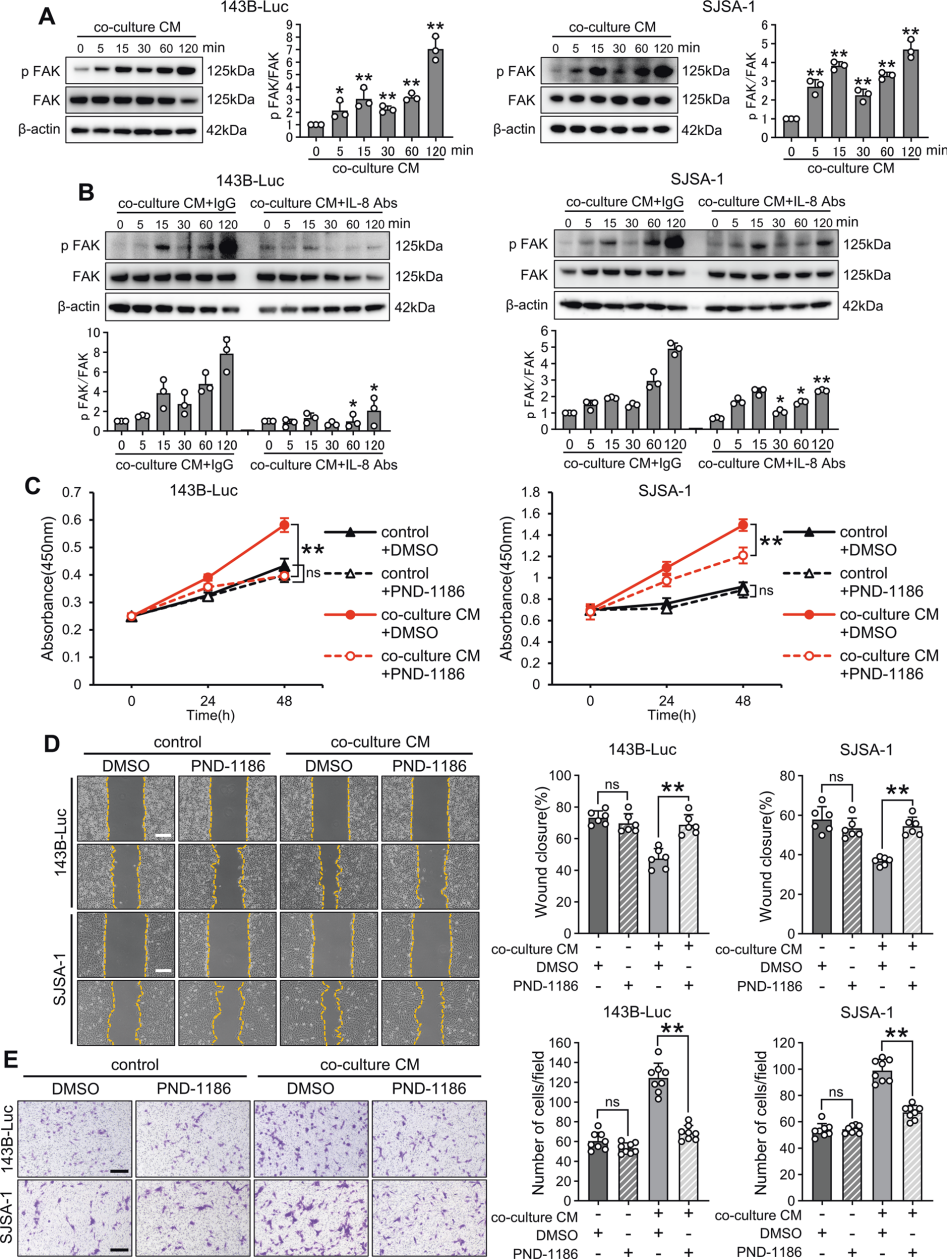
Role of the FAK pathway in IL-8 production in co-culture CM. **A** Western blot assays for FAK phosphorylation in OS cells treated with co-culture CM and quantification of western blot bands. **B** Western blot assays for the phosphorylation of FAK (p FAK) in OS cells treated with co-culture CM + isotype IgG (IgG) or + anti-IL-8 antibodies (IL-8 Abs, 1 µg/mL) and quantification of western blot bands. The FAK phosphorylation levels were also compared between the same times. **C** Cell viability assay for OS cells treated with or without co-culture CM and with or without FAK inhibitor (PND-1186, 1 µM) using Cell Counting Kit-8. **D**, **E** Scratch and invasion assay for OS cells treated with or without co-culture CM and with or without FAK inhibitor (PND-1186) (1 µM). The scratch assay was performed for 12 h, and the invasion assay was conducted for 24 h. Scale bars represent 500 μm. CM conditioned medium, FAK focal adhesion kinase, ns not significant, OS osteosarcoma. Data are presented as mean ± standard deviation. *p < 0.05 and **p < 0.01. All data were obtained from at least three independent experiments.

Given the significance of the IL-8–FAK pathway in cancer [12], we hypothesized that this pathway might exert similar effects on OS proliferation, migration, and invasion. Concordantly, FAK phosphorylation was observed in both OS cell types upon exposure to recombinant IL-8 (Supplemental Fig. 4A). Monitoring the phosphorylation of FAK in co-culture CM revealed a bimodal peak, similar to the response observed with recombinant IL-8 (Fig. 5A). Further, the addition of an anti-IL-8 antibody to co-culture CM substantially inhibited FAK phosphorylation (Fig. 5B). Finally, using FAK phosphorylation inhibitors diminished the promoting effects of co-culture CM (Fig. 5C–E). Collectively, our findings suggest that the IL-8 within co-culture CM augments OS proliferation, migration, and invasion through the FAK pathway.

### IL-8 within co-culture CM promotes OS proliferation and metastasis in vivo

**Fig. 6:**
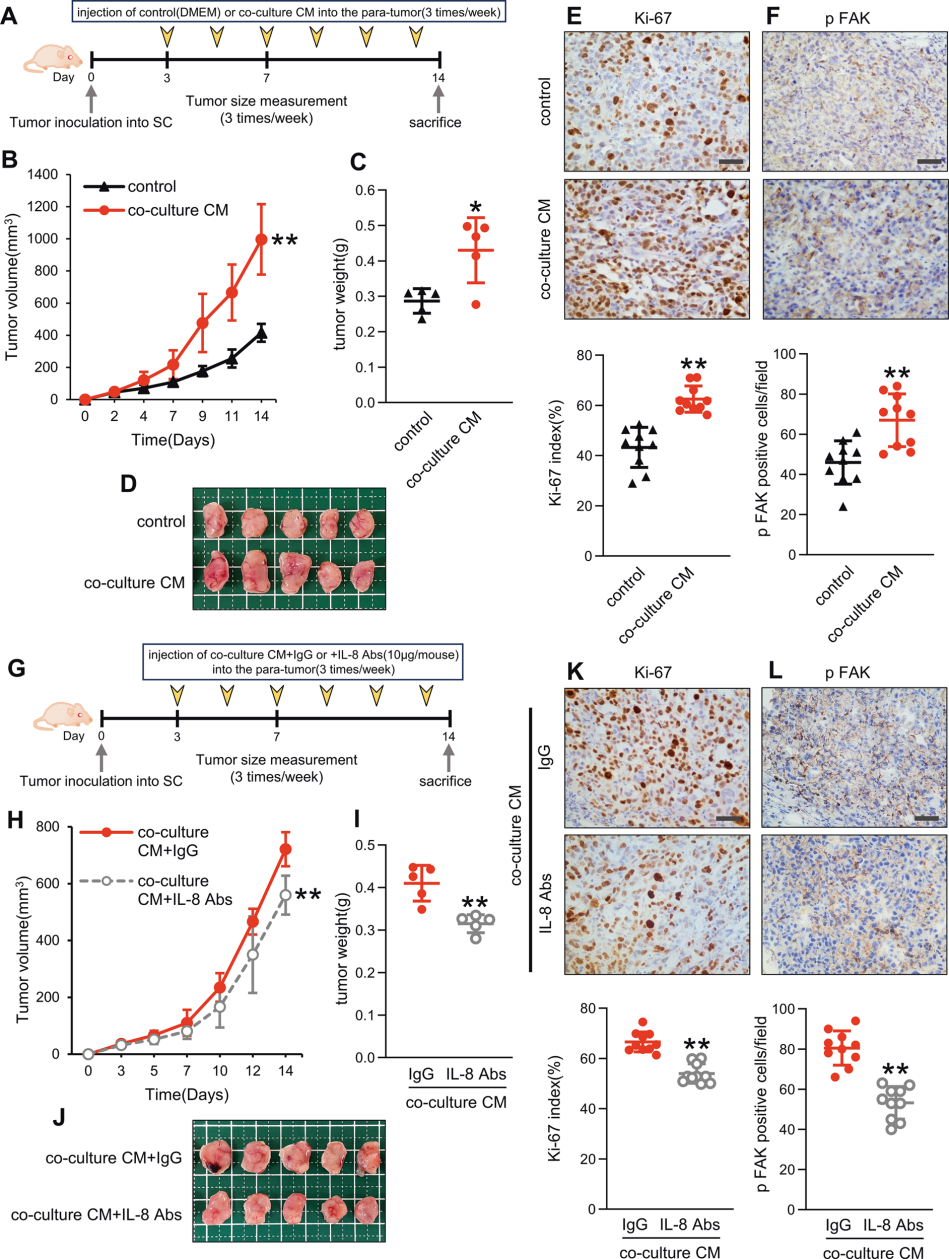
Effect of IL-8 in co-culture CM on primary growth. **A** Schematic showing the experimental design for subcutaneous transplantation of OS cells. 143B-Luc cells (2 × 10^6^/mouse) were subcutaneously transplanted in 6–7-week-old BALB/c nu/nu mice, and DMEM or co-culture CM was injected into the para-tumor thrice per week. **B** Tumor volume with or without co-culture CM measured thrice per week after tumor transplantation. **C**, **D** Tumor weight and image of the excised tumor with or without co-cultured CM 14 days after tumor transplantation. **E**, **F** Immunohistochemistry and quantification of Ki-67 and phospho-FAK in tumor sections with or without co-culture CM 14 days after tumor transplantation. Two fields of view per sample were randomly selected, and quantification was performed in 10 fields. **E** Ki-67-positive cells in the field were counted and indicated as Ki-67 labeling index. Scale bars represent 50 μm. **G** Schematic showing the experimental design for subcutaneous OS transplantation using anti-IL-8 antibodies (IL-8 Abs). **H** Tumor volume after pretreatment with co-culture CM with or without IL-8 Abs (10 µg/mouse) measured thrice per week after tumor transplantation. **I** and **J** Weight and image of the excised tumor pretreated with co-culture CM with or without IL-8 Abs 14 days after tumor transplantation. **K**, **L** Immunohistochemistry and quantification of Ki-67 and phospho-FAK in tumor sections with co-culture CM with or without IL-8 Abs 14 days after tumor transplantation. Scale bars represent 50 µm. CM conditioned medium, DMEM Dulbecco’s modified Eagle’s medium, FAK focal adhesion kinase, OS osteosarcoma. Data are presented as mean ± standard deviation; *p < 0.05, **p < 0.01. Each group contained five animals.

We investigated its effects of IL-8 within co-culture CM on the primary site by subcutaneously transplanting OS cells in nude mice and injecting co-culture CM into the para-tumor area thrice per week (Fig. 6A). In the co-culture CM group, 143B-Luc and SJSA-1 cells substantially increased the tumor volume and weight (Fig. 6B–D, Supplemental Fig. 5A-C). IHC analysis revealed higher Ki-67 index and phospho-FAK levels in the co-culture CM group than that in the control group (Fig. 6E, F).

Introducing anti-IL-8 antibody into co-culture CM in the same subcutaneous xenograft tumor model (Fig. 6G) suppressed the tumor volume and weight of both 143B-Luc and SJSA-1 cells in the anti-IL-8 antibody group (Fig. 6H–J, Supplemental Fig. 5D–F) compared with that in the control group. Notably, the anti-IL-8 antibody did not induce weight loss in the mice (Supplemental Fig. 5G). Ki-67 index and phospho-FAK were reduced in the anti-IL-8 antibody group (Fig. 6K, L).

**Fig. 7:**
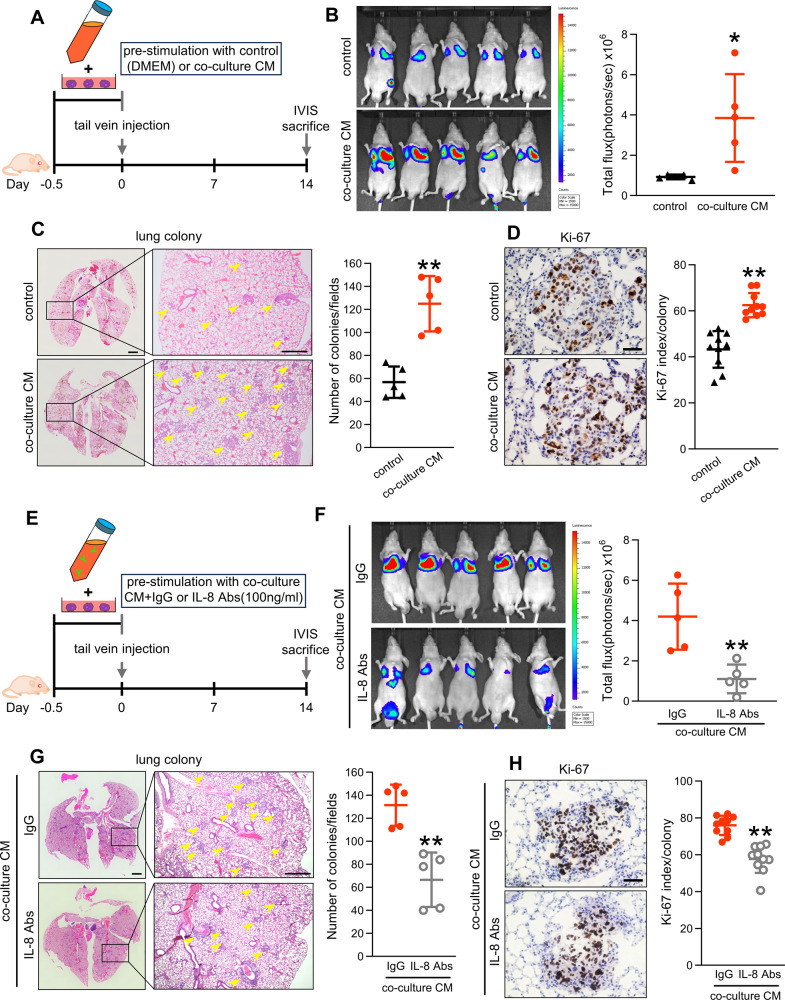
Effect of IL-8 in co-culture CM on lung metastasis. **A** Schematic showing the experimental design for OS tail vein injection. 143B-Luc cells were treated with DMEM or co-culture CM for 12 h prior to tail vein injection; 143B-Luc cells (1 × 10^6^/mouse) were injected into the tail vein of 6–7 week-old BALB/c nu/nu mice. **B** IVIS imaging and quantification of lung metastasis 14 days after the injection of tumors pretreated with or without co-culture CM. **C** Hematoxylin and eosin staining of lung sections with or without co-culture CM and quantification of lung colonies. Scale bars represent 1 mm and 500 µm. **D** Immunohistochemistry of Ki-67 positive cells in lung colonies with or without co-culture CM and quantification of the Ki-67 labeling index. Two colonies per sample were randomly selected, and quantification was performed for 10 colonies. Ki-67-positive cells in the colonies were counted and shown as the Ki-67 labeling index. Scale bars represent 100 µm. **E** Schematic showing the experimental design for OS tail vein injection using anti-IL-8 antibody. **F** IVIS imaging and quantification of lung metastasis 14 days after the injection of tumors pretreated with co-culture CM with or without IL-8 antibodies. **G** Hematoxylin and eosin staining of lung sections with co-culture CM with or without IL-8 Abs and quantification of lung colonies. Scale bars represent 1 mm and 500 µm. **H** Immunohistochemistry of Ki-67-positive cells in lung colonies with co-culture CM with or without IL-8 Abs and quantification of Ki-67 labeling index. Scale bars represent 100 µm. CM conditioned medium, DMEM Dulbecco’s modified Eagle’s medium, IVIS, in vivo imaging system, OS osteosarcoma. Data are presented as mean ± standard deviation; *p < 0.05, **p < 0.01. Each group contained five animals.

We further investigated its effect on lung metastasis, which is a critical factor in OS prognosis by pre-treating OS cells with co-culture CM and intravenously injecting it into mouse tails (Fig. 7A). After 14 days, IVIS, lung colony counts, and Ki-67 index were all higher in the co-culture CM group than that in the control group (Fig. 7B–D). The metastasis-promoting effect of co-culture CM was mitigated by adding anti-IL-8 antibody to co-culture CM (Fig. 7E–H), suggesting that IL-8 within co-culture CM promotes proliferation and metastasis in vivo, thereby highlighting the potential of IL-8 as a novel therapeutic target.

## Discussion

The crosstalk between tumor and stromal cells, including immune cells such as platelets and macrophages, within the TME fosters a tumor-promoting environment. We previously confirmed the involvement of the coagulation system and platelets, a constituent of the TME, in OS proliferation and metastasis [13, 14]; however, the significance of TAMs remained predominantly unexplored in this context. Our findings revealed a higher abundance of TAMs in lung metastatic sites than that in primary tumor sites in patient samples. We demonstrated the role of IL-8, a TAM-derived cytokine, in promoting OS proliferation and metastasis via the IL-8–FAK axis. Furthermore, we validated the effect of TAM-derived IL-8 for the first time in a mouse model, at primary tumor and metastatic sites. Collectively, our findings provide valuable insights for the development of novel therapies targeting IL-8 within TAMs and the broader TME.

We previously reported that CD163-positive macrophages exert a tumor-promoting effect in undifferentiated pleomorphic sarcoma, with high CD163 expression associated with shorter overall survival [8]. Conversely, reports on OS have indicated that higher CD163^+^TAMs are associated with better prognosis; however, this relationship is inconsistent [15]. Our present findings revealed increased CD163^+^TAMs in metastatic lesions than that in primary tumors, suggesting a potential prognostic significance. Further investigations are required to elucidate the precise role of CD163 in OS. Moreover, there may exist other sarcoma-specific markers, warranting further gene expression analyses using techniques such as microarrays.

We reported the tumor-promoting effects of TAM-derived cytokines in carcinomas [5]. While sarcomas exhibit the tumor-promoting effects of CCL18 [16] and IL-6 [8], the impact of other cytokines remains unclear. We elucidated the cytokine landscape within TAMs in OS by revealing the substantial increase in the IL-8 levels. Furthermore, single-cell analyses revealed the potential for Iba-1^+^CD163^+^TAMs to express more IL-8, highlighting the importance of IL-8 within the TME.

IL-8, a prototypical cytokine belonging to the CXC family, primarily binds to G protein-coupled receptors CXCR1 and CXCR2 [17], and induces inflammation via neutrophils and granulocytes [18]. In carcinomas, IL-8 is associated with PI3K and PKC pathway-mediated tumor-promoting effects [19]. The effects of TAM-derived IL-8, such as estrogen receptor suppression in uterine cancer [20] and epithelial–mesenchymal transition (EMT) induction in oral cancer [21], have been reported in carcinomas. On the other hand, in OS, self-seeding circulating tumor cells have been reported to produce IL-8, which promotes tumor growth and metastasis [22]. While the significance of IL-8 in OS is becoming clearer, the effects of TAM-derived IL-8 in sarcomas have remained elusive. Our study is the first to establish that TAM-derived IL-8 directly influences OS growth and metastasis both in vitro and in vivo. Notably, OS stimulated by TAM-derived IL-8 acquired lung metastatic ability, suggesting that anti-IL-8 antibodies may not only shrink primary tumors but also prevent metastasis. Further research is warranted to explore these possibilities.

The IL-8–CXCR1/2 axis activates various signaling pathways, including PI3K and PKC [18, 23], one of which is the FAK pathway [9]. FAK is a non-receptor tyrosine kinase that governs cell motility and survival [24]. It undergoes autophosphorylation in response to signals from integrins and growth factors [25]. Increased expression of FAK and its phosphorylated counterpart has been observed in cervical [26] and lung [27] cancers. In OS, FAK is recognized as an essential player, with its downregulation associated with reduced migration and invasion, and its expression and phosphorylation status serving as prognostic indicators [28]. Concerning the IL-8-FAK axis in OS, reports had hinted at the involvement of mesenchymal stem cell-derived IL-8 [29], though the precise role remained unclear. We demonstrated that IL-8 impacts proliferation, migration, and invasion, with these effects being suppressed by FAK inhibitors. Currently, FAK inhibitors are undergoing clinical trials in various cancer types, including glioblastoma [30] and meningioma [31]. Our findings indicate that among the multiple pathways activated by IL-8, FAK plays a role in promoting proliferation, migration, and invasion, potentially serving as a therapeutic target. Future investigations may reveal even more contribution of various other pathways, such as PI3K and PKC, beyond FAK.

Despite the use of established anticancer drugs, such as methotrexate and ifomide, in OS treatment, no new regimens have emerged, and survival rates have plateaued for the past two decades [32]. A comprehensive understanding of lung metastasis mechanisms and anticancer drug resistance would directly translate into improved prognoses. In clinical practice, when recurrence or metastasis occurs after completion of treatment, patients often face drug resistance or the need to reduce drug doses owing to hepatic or renal impairments. Accordingly, we selected IL-8 within the TME for this study, as clinical trials involving IL-8 receptor inhibitors and anti-IL-8 antibodies have been conducted in breast [33] and prostate [34] cancers. Expanding the indication of these drugs to sarcomas, including OS, can potentially help reduce adverse events such as kidney and liver damage when used in conjunction with existing first-line therapies. This broader application could provide treatment for cases where first-line therapies prove ineffective, thereby enhancing the range of available treatments.

There are several limitations to this study. First, the factors triggering the production of IL-8 by OS and TAMs remain unclear. As IL-8 expression is induced by various stimuli, including cytokines (such as IL-1 and TNFα), as well as environmental stressors and transcription factors (such as NF-κB) [35–37], identifying potential triggers is essential for a more effective inhibition. Second, IL-8 is not naturally expressed in mice. Therefore, confirming IL-8 production from mouse macrophages on the host side is challenging. IL-8 has physiological effects on other TME constituents, such as neutrophils, and the suppressive impact of TAM-derived IL-8 on CD8+ T cells [38]. Therefore, future research using IL-8-knock-in transgenic mice is warranted. Third, IL-8 could not be detected in the IHC of patient samples, possibly owing to the influence of demineralization [39].

Nevertheless, we identified IL-8 as a cytokine produced within the TME created by interactions between OS and TAMs. We further confirmed its significance in vitro, in vivo, and in patient samples. Additionally, our findings highlighted the IL-8–FAK axis as a critical pathway through which IL-8 affects OS cells. Considering the stagnant development of chemotherapy for OS, IL-8 emerges as a promising novel therapeutic target.

## Materials and methods

### OS tissue samples and patient data

We included five patients who underwent surgery for both the primary site and lung metastasis of OS at the hospitals of the University of Yamanashi, Shinshu University, and Saitama Medical University (Table 1). The study was approved by the ethics committees of each institution (No. 2456). Informed consent for using samples and clinical data was obtained from patients or their legal guardians.

### Immunohistochemistry

All patients had multiple lung metastases (five or fewer). Among the five patients, no distinct morphological differences between each tumor colony were identified. Therefore, representative samples, which were selected based on tumor size and viability under appropriate conditions, were examined using immunohistochemistry. IHC for Iba1 and CD163 was performed as previously described [8]. Iba1- and CD163-positive cells were quantified in ten randomly selected areas of a high-power microscope field. Double immunostaining of Iba1 and CD163 was performed following established methods [40]. IHC staining was performed using primary antibodies against Iba1 (013-27691, 1:1000; Fujifilm, Tokyo, Japan) and CD163 (CD163-L-CE, 1:500; Leica Biosystems, Nussloch, Germany). Subsequently, the samples were incubated with horseradish peroxidase-labeled secondary anti-mouse antibody (Nichirei, Tokyo, Japan). The reaction was visualized using the diaminobenzidine system (Nichirei). For double-IHC, sections were subjected to microwave treatment, incubated with anti-Iba-1 antibody, and visualized using HistoGreen (Linaris, Heidelberg, Germany). The staining area and size was evaluated using Image J software (Wayne Rasband, National Institutes of Health, Bethesda, MD, USA). For the analysis of infiltrating macrophages, ten non-overlapping high-power fields (×400 magnification) were randomly selected in tumor areas without necrosis, hemorrhage, and osteoid matrix, and the proportion (%) of positive areas was evaluated using the ImageJ software (Supplemental Fig. 6). For cell size analysis, 100–150 cells per sample that had their nucleus in the pictures were encircled, and the size of encircled area was measured based on the scale bar (Supplemental Fig. 6).

### Cell culture and reagents

Human OS cell lines (143B, SJSA-1, HOS, and U2OS) were obtained from ATCC (American Type Culture Collection, VA, USA). 143B-Luc cells, which fluoresce in the presence of the d-luciferin substrate, were generated by transfecting 143B cells with the lentiviral luciferase reporter plasmid as previously described [14]. OS cells were cultured in Dulbecco’s modified Eagle’s medium (DMEM) (Thermo Fisher Scientific, CA, USA) supplemented with 10% heat-inactivated fetal bovine serum (FBS) (Thermo Fisher Scientific) and 1% penicillin and streptomycin (Thermo Fisher Scientific) at 37 °C under 5% CO_2_ conditions. Cells were passaged twice a week, and morphology was periodically monitored. Human leukemic THP-1 monocytes, obtained from RIKEN Cell Bank (Ibaraki, Japan), were cultured in Roswell Park Memorial Institute 1640 medium (RPMI) (Thermo Fisher Scientific) supplemented with 10% FBS, 1% penicillin and streptomycin, and 0.05 mM 2-mercaptoethanol (#1610710, BIORAD, CA, USA) at 37°C in a 5% CO_2_ environment. Peripheral blood mononuclear cells (PBMCs) were isolated from freshly drawn blood using density gradient centrifugation with SepMate columns (StemCell Technologies, Vancouver, Canada) following the manufacturer’s instructions. The reagents used were recombinant IL-8 (208-IL, R&D, MN, USA), mouse IgG1 isotype control (MAB002, R&D), anti-IL-8 antibody (MAB208, R&D), dimethyl sulfoxide (DMSO) (D8418, Sigma-Aldrich, Darmstadt, Germany), and PND-1186 (S7653, Selleck, TX, USA). All cell cultures were tested for *Mycoplasma* contamination every 3 months using the MycoAlert Mycoplasma Detection Kit (Lonza, MI, USA).

### CM preparation

THP-1 cells were cultured in 100 ng/mL phorbol 12-myristate 13-acetate (PMA) (P8139, Sigma-Aldrich) for 48 h to induce macrophage differentiation. PBMCs were cultured in 50 ng/mL recombinant M-CSF (300-25, PeproTech, NJ, USA) and 2% human serum for 6 days to promote macrophage differentiation (human monocytes derived macrophage [HMDMs]). After washing thrice with phosphate-buffered saline (PBS) (Thermo Fisher Scientific), the cells were co-cultured in an OS: macrophage ratio of 2:1 for 24 h, and the supernatant was collected (co-culture CM). Subsequently, the differentiated macrophages were cultured in DMEM + 10% FBS for 24 h, and the supernatant of macrophages alone was collected (macrophage CM). OS cells were cultured in DMEM + 10% FBS for 24 h after seeding, and the supernatant of OS was collected (OS-CM).

### Cytokine array

Cytokine array analysis was performed using a human cytokine array kit (Human Cytokine Antibody Array C5, RayBiotech, GA, USA), following the manufacturer’s protocol. Images were captured using a ChemiDoc Touch (BIORAD).

### ELISA

IL-8 concentrations in the cell culture supernatants were measured using a commercially available IL-8 ELISA kit (Biolegend, CA, USA), following the manufacturer’s instructions. The absorbance was measured at 450 nm using an SH-1100R microplate reader (Corona Electric Co., Ibaraki, Japan).

### Quantitative real-time PCR

Total RNA was extracted from OS and macrophage cells using the RNeasy mini kit and treated with DNase (Qiagen, Venlo, Netherlands). Purity of the RNA samples was assessed spectrophotometrically by measuring the ratio of the optical density at 260 nm and 280 nm (OD260/280). The reverse transcription was performed immediately following the quality control assessment as previously described [14]. The primers used were IL-8 (Hs00174103_m1, Thermo Fisher Scientific) and hypoxanthine phosphoribosyl transferase (Hs02800695_m1, Thermo Fisher Scientific). Quantitative PCR analysis was performed using the StepOnePlus real-time PCR system (Applied Biosystems, MA, USA). The thermal cycling conditions were as follows: 95 °C for 20 s followed by 40 cycles of 95 °C for 1 s and 60 °C for 20 s.

### Single-cell RNA-seq data

Single-cell RNA-seq data of 11 OS cases, including two lung metastatic cases, from the original report [41] were used in the analysis. The R package Seurat was employed for the analysis. Initially, quality control was performed on all 11 datasets. High-quality cells were defined as those expressing over 500 and fewer than twice the median of the detected genes in each cell. The proportion of mitochondrial genes expressed in high-quality cells was required to be <10%. The 11 datasets were then loaded and merged into a single Seurat object. The data were normalized using the *LogNormalize* function. The top 3000 highly variable genes were used for further analysis. The data were scaled for all genes, and principal component analysis (PCA) was performed. The R package harmony was used to reduce batch effects. The *ElbowPlot* function was employed to determine the most informative principal components of the PCA for the harmony object. The top 50 components were used for further analysis. The cells were clustered with a resolution of 0.4 using the FindClusters function, resulting in the identification of 27 clusters. Genes differentially expressed between clusters were identified using the *FindAllMarkers* function. Myeloid cells in clusters 1, 8, 13, 15, and 22 were identified based on the method in the original report [41] and their high Iba1 expression. The myeloid cell group was re-clustered with a resolution of 1 into 19 clusters, and genes differentially expressed between clusters were identified using the *FindAllMarkers* function. The cells were initially divided into CD163^+^ (0, 1, 3, 5, 10, 11, 15, 16, 18) and CD163^−^ (2, 6, 7, 8, 9, 12, 13) groups. Clusters 7 and 12, characterized by high CD163 and CD1C expression, respectively, served as dendritic cells (DCs), whereas clusters 5 and 18, featuring high MKI67 expression, were identified as CD163^+^ proliferating cells. IL-8 expression in two lung metastatic cases across different cell types were summarized using violin plots.

### Western blotting

OS cells were collected, and western blotting was performed as previously described [13]. Blots were blocked in TBS with 0.05% Tween 20 (Sigma-Aldrich) buffer (TBS-T) containing 1% Bovine Serum Albumin (BSA) (013-27054, Fujifilm), followed by incubation in TBS-T with 1% BSA and primary antibodies. Primary antibodies used were anti-FAK antibody (#13009, 1:1000, Cell Signaling Technology, MA, USA), anti-phospho-FAK antibody (44-624G, 1:1000, Invitrogen, MA, USA), anti-CXCR1 antibody (BS60349, 1:500, Bioworld, Bloomington, MN, USA), anti-CXCR2 antibody (20634-1 AP, 1:1000, Proteintech, IL, USA), and anti-β-actin antibody (A2228, 1:1000, Sigma-Aldrich). After washing thrice with TBS-T, membranes were incubated with secondary antibodies. Secondary antibodies used were anti-rabbit IgG antibody (#7074, 1:2000, Cell Signaling Technology) and anti-mouse IgG antibody (#7076, 1: 2000, Cell Signaling Technology). Images were captured using a ChemiDoc Touch and quantified using Image J software.

### Cell viability assay

OS cells were seeded at 4 × 10^3^ cells in 96-well plates with 200 μL of DMEM containing 1% FBS. Stimulation with recombinant IL-8, co-culture CM, anti-IL-8 antibody, or PND-1186 was repeated every 24 h. Cell viability was assessed using a Cell Counting Kit-8 (WST-8; Dojindo Molecular Technologies, Inc., Kumamoto, Japan) according to the manufacturer’s instructions at 0, 24, and 48 h. The absorbance of the cultures was measured at 450 nm using an SH-1100R microplate reader.

### Scratch wound healing assay

OS cells were seeded in a 10-cm petri dish, and after cell adhesion, stimulation was performed for 12 h with or without recombinant IL-8, co-culture CM, anti-IL-8 antibody, or PND-1186. OS cells were seeded at 70 × 10^4^ in a 6-well plate 12-h post-stimulation. When they adhered and reached 90% confluence, the cells were scratched using a 200-µL tip to create a wound, and the medium was changed to serum-free DMEM (i.e., with 0% FBS). After 12 h, the migration area was evaluated by measuring the cell-free area using Image J software. The percent wound area was calculated from the cell-free area after introducing the scratch (0 h) and that after 12 h (12 h) using the formula:

$${\rm{percent\; wound\; area}}=[(0{\rm{h}}-12{\rm{h}})/0{\rm{h}}]\times 100$$

### Cell invasion assay

OS cells were stimulated with or without recombinant IL-8, co-culture CM, anti-IL-8 antibody, or PND-1186 for 24 h before the assay. 143B-Luc (2 × 10^3^) or SJSA-1 (6 × 10^3^) cells were seeded onto Chemotaxicell filters with a pore size of 8 µm (CH8-24, Kurabo, Tokyo, Japan) that had been coated with 300 µg/mL Atelocollagen (IAC-30, KOKEN CO., Tokyo, Japan), and the invasion assay was performed as previously described [42]. After 24 h, invading cells per field of view were counted under an optical microscope.

### Mouse

Male BALB/c nude mice (6–7-week-old; CLEA Japan, Tokyo, Japan) were housed under standard conditions with a 12-h light/dark cycle and provided with adequate water and food. Animal experiments were conducted following the Guidelines for Proper Conduct of Animal Experiments, Science Council of Japan. Protocols were approved by the Animal Care and Use Committee (No. A4-13) of the University of Yamanashi.

### In vivo tumorigenesis

To investigate the effect of co-culture CM on the primary tumor, OS cell lines (2 × 10^6^ 143B-Luc and SJSA-1 cells per 100 μL) were subcutaneously transplanted into the dorsal side of mice. After 3 days, DMEM and co-culture CM were injected near the tumor in the control and co-culture CM groups, respectively (n = 5 each; 100 μL each, thrice per week).

Anti-IL-8 antibodies (10 µg/mouse) were mixed with co-culture CM and injected subcutaneously near the tumor. Tumor volume (length × width^2^ /2) was measured thrice a week. Two weeks after subcutaneous implantation, mice were sacrificed with deep anesthesia, and tumor weights were measured.

To assess the effect of co-culture CM on lung metastasis, 143B-Luc cells were stimulated with DMEM with 10% FBS (control group, n = 5) or co-culture CM (co-culture CM group, n = 5) for 12 h before tail vein injection. 143B-Luc cells (1 × 10^6^ cells/100 μL) were injected into the tail vein of mice. Anti-IL-8 antibodies and co-culture CM were mixed to investigate the potential inhibition of tumor metastasis. Two weeks later, luciferin (E1602, Promega, WI, USA) was intravenously administered into the retro-orbital vein of anesthetized mice using isoflurane. The emission intensity was measured using the IVIS Lumina imaging system (SPI Engineering Co., Ltd., Nagano, Japan). Mice were randomly allocated to experimental groups and no blinding method was used for injection. There was no animal exclusion criteria.

### Immunohistochemistry of murine samples

IHC staining was performed on tumor or lung samples resected on day 14 post-inoculation of OS cells, as previously described [42]. IHC staining was performed using primary antibodies against Ki-67 (ab16667, 1:200; Abcam, Cambridge, UK) and phospho-FAK (Tyr397) (44-624 G, 1:1000; Invitrogen).

### Statistical analysis

Data are presented as means (±standard deviation) from at least three independent experiments. No statistical methods were used to predetermine the sample size. Significance was determined using the Student’s or Welch’s t-test after an F-test was performed. If the raw data did not fit a normal distribution, the Mann–Whitney U-test was used for analysis. The variance was similar between the groups that were being statistically compared. Statistical significance was set at p < 0.05 (* represents p < 0.05; ** represents p < 0.01).

### Reporting summary

Further information on research design is available in the Nature Research Reporting Summary linked to this article.

### Supplementary information

Supplemental Fig.1

Supplemental Fig.2

Supplemental Fig.3

Supplemental Fig.4

Supplemental Fig.5

Supplemental Fig.6

Original western blots

Reporting Summary

## Data Availability

All the data presented in this study are available from the corresponding author upon request.
