# Correction: Pivotal role of IL-8 derived from the interaction between osteosarcoma and tumor-associated macrophages in osteosarcoma growth and metastasis via the FAK pathway

**DOI:** 10.1038/s41419-024-06795-3

**Published:** 2024-07-02

**Authors:** Rikito Tatsuno, Jiro Ichikawa, Yoshihiro Komohara, Cheng Pan, Tomonori Kawasaki, Atsushi Enomoto, Kaoru Aoki, Keiko Hayakawa, Shintaro Iwata, Takahiro Jubashi, Hirotaka Haro

**Affiliations:** 1 Department of Orthopaedic Surgery, University of Yamanashi, Yamanashi, Japan; 2 Department of Cell Pathology, Graduate School of Medical Sciences, Kumamoto University, Kumamoto, Japan; 3 Department of Pathology, Saitama Medical University International Medical Center, Saitama, Japan; 4 Department of Pathology, Nagoya University Graduate School of Medicine, Aichi, Japan; 5 Physical Therapy Division, School of Health Sciences, Shinshu University, Nagano, Japan; 6 Department of Orthopaedic Oncology, Cancer Institute Hospital of Japanese Foundation for Cancer Research, Tokyo, Japan; 7 Department of Musculoskeletal Oncology and Rehabilitation, National Cancer Center Hospital, Tokyo, Japan

**Keywords:** Bone cancer, Cancer microenvironment

Correction to: *Cell Death and Disease* 10.1038/s41419-024-06487-y, published online 01 February 2024

The graph presented in the original Supplementary figure 4 was incorrect and has been replaced by a correct version. The original article has been corrected.

### Supplementary information

Corrected Supplemental Fig. 4

