# Supplementary material for: Pivotal role of IL-8 derived from the interaction between osteosarcoma and tumor-associated macrophages in osteosarcoma growth and metastasis via the FAK pathway
Source: Cell Death Dis. 2024 Feb 1;15(2):108. doi: 10.1038/s41419-024-06487-y (PMC10834992; doi:10.1038/s41419-024-06487-y)

Original western blots

CXCR1 (43kDa) (Supple. 3A)
143B-Luc and SJSA-1

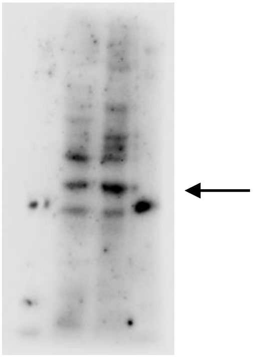

CXCR2 (45-50kDa) (Supple. 3A)
143B-Luc and SJSA-1

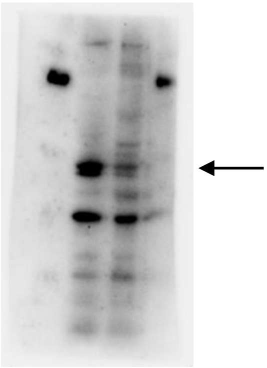

β-actin (42kDa) (Supple. 3A)
143B-Luc and SJSA-1

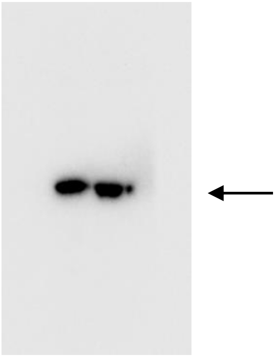

phospho-FAK (125kDa)
(Figure. 5A) 143B-Luc

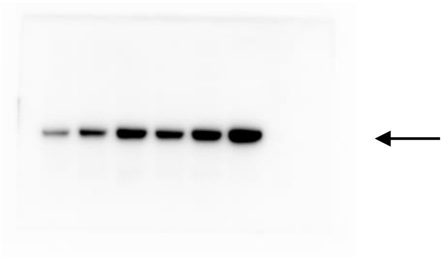

phospho-FAK (125kDa)
(Figure. 5A) SJSA-1

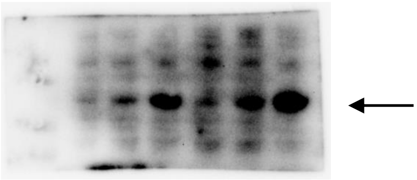

FAK (125kDa)
(Figure. 5A) 143B-Luc

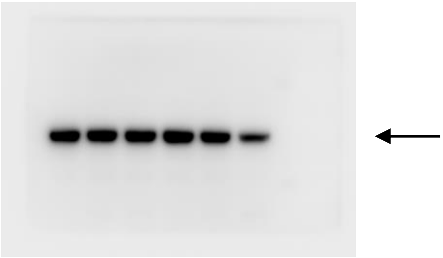

FAK (125kDa)
(Figure. 5A) SJSA-1

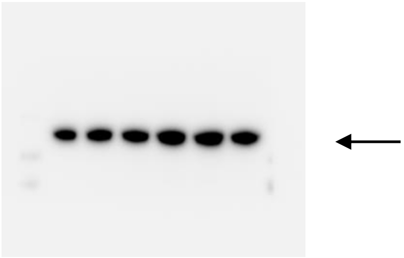

β-actin (42kDa)
(Figure. 5A) 143B-Luc

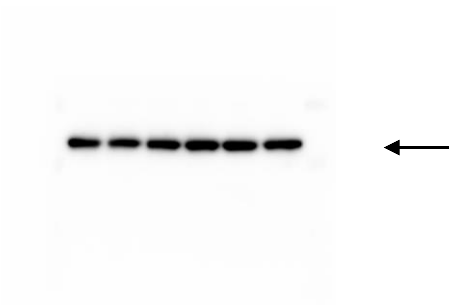

β-actin (42kDa)
(Figure. 5A) SJSA-1

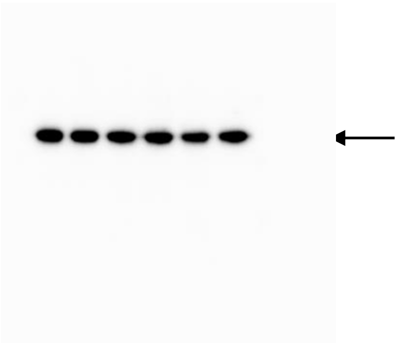

phospho-FAK (125kDa)
(Figure. 5B) 143B-Luc

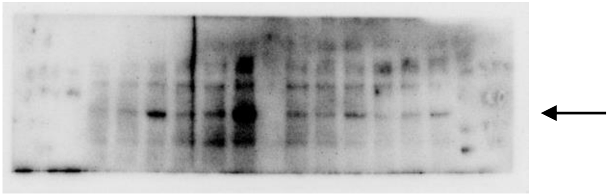

FAK (125kDa)
(Figure. 5B) 143B-Luc

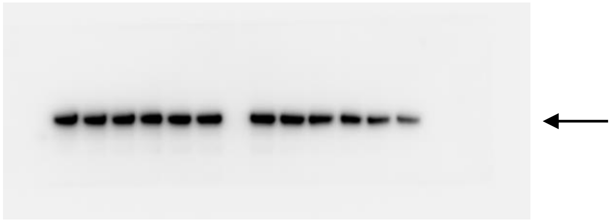

β-actin (42kDa)
(Figure. 5B) 143B-Luc

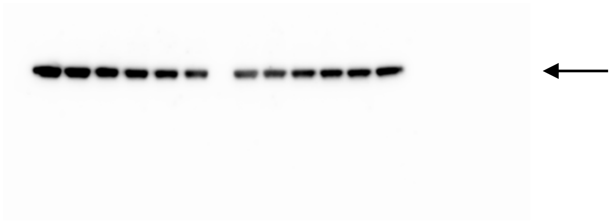

phospho-FAK (125kDa)
(Figure. 5B) SJSA-1

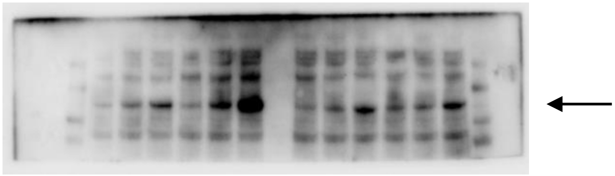

FAK (125kDa)
(Figure. 5B) 143B-Luc

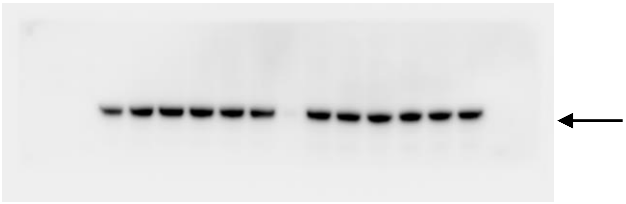

β-actin (42kDa)
(Figure. 5B) 143B-Luc

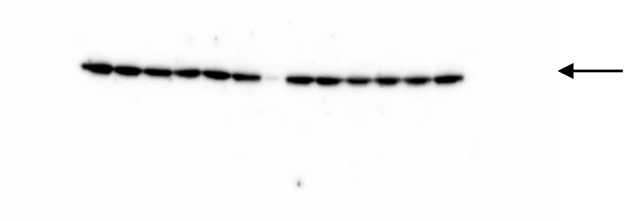

phospho-FAK (125kDa)
(Supple. 4A) 143B-Luc

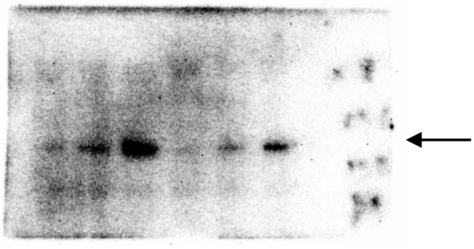

phospho-FAK (125kDa)
(Supple. 4A) SJSA-1

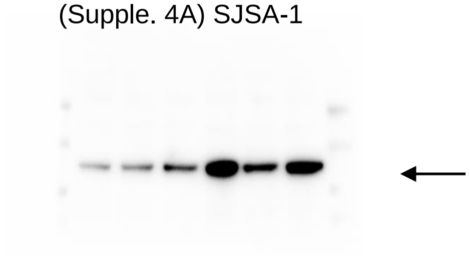

FAK (125kDa)
(Supple. 4A) 143B-Luc

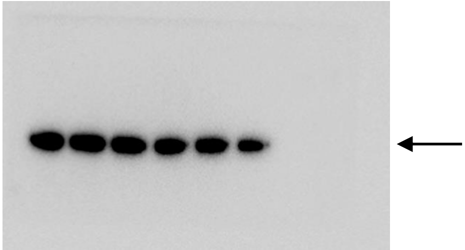

FAK (125kDa)
(Supple. 4A) SJSA-1

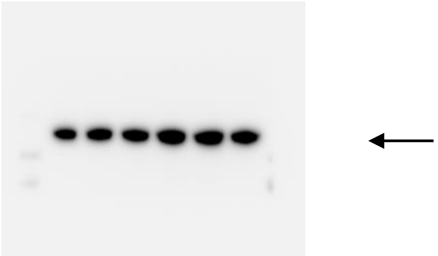

β-actin (42kDa)
(Supple. 4A) 143B-Luc

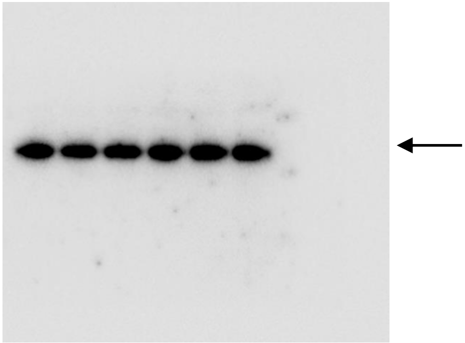

β-actin (42kDa)
(Supple. 4A) SJSA-1

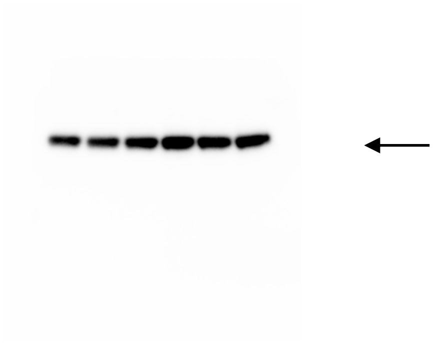

Supplement: Supplementary file 7 — Original western blots [file 41419_2024_6487_MOESM7_ESM.pdf]
